# Gene Expression Profiling and Pathway Network Analysis Predicts a Novel Antitumor Function for a Botanical-Derived Drug, PG2

**DOI:** 10.1155/2015/917345

**Published:** 2015-04-20

**Authors:** Yu-Lun Kuo, Chun-Houh Chen, Tsung-Hsien Chuang, Wei-Kai Hua, Wey-Jinq Lin, Wei-Hsiang Hsu, Peter Mu-Hsin Chang, Shih-Lan Hsu, Tse-Hung Huang, Cheng-Yan Kao, Chi-Ying F. Huang

**Affiliations:** ^1^Department of Computer Science and Information Engineering, National Taiwan University, No. 1, Section 4, Roosevelt Road, Taipei 10617, Taiwan; ^2^Institute of Statistical Science, Academia Sinica, No. 128, Section 2, Academia Road, Nankang, Taipei 11529, Taiwan; ^3^Immunology Research Center, National Health Research Institutes, No. 35, Keyan Road, Zhunan, Miaoli County 35053, Taiwan; ^4^Institute of Biopharmaceutical Sciences, National Yang-Ming University, No. 155, Section 2, Linong Street, Taipei 11221, Taiwan; ^5^Institute of Clinical Medicine, National Yang-Ming University, No. 155, Section 2, Linong Street, Taipei 11221, Taiwan; ^6^Division of Hematology and Oncology, Department of Medicine, Taipei Veterans General Hospital, No. 201, Section 2, Shipai Road, Beitou, Taipei 11217, Taiwan; ^7^Department of Education and Research, Taichung Veterans General Hospital, No. 1650 Taiwan Boulevard Section 4, Taichung 40705, Taiwan; ^8^Department of Traditional Chinese Medicine, Chang Gung Memorial Hospital, No. 222, Maijin Road, Keelung 20401, Taiwan; ^9^Graduate Institute of Clinical Medicine Sciences, Chang Gung University, No. 259, Wenhua 1st Raod, Taoyuan 33302, Taiwan; ^10^Graduate Institute of Traditional Chinese Medicine, Chang Gung University, No. 259, Wenhua 1st Road, Taoyuan 33302, Taiwan; ^11^Graduate Institute of Biomedical Electronic and Bioinformatics, National Taiwan University, No. 1, Section 4, Roosevelt Road, Taipei 10617, Taiwan; ^12^Department of Biotechnology and Laboratory Science in Medicine, National Yang-Ming University, No. 155, Section 2, Linong Street, Taipei 11221, Taiwan

## Abstract

PG2 is a botanical drug that is mostly composed of *Astragalus* polysaccharides (APS). Its role in hematopoiesis and relieving cancer-related fatigue has recently been clinically investigated in cancer patients. However, systematic analyses of its functions are still limited. The aim of this study was to use microarray-based expression profiling to evaluate the quality and consistency of PG2 from three different product batches and to study biological mechanisms of PG2. An integrative molecular analysis approach has been designed to examine significant PG2-induced signatures in HL-60 leukemia cells. A quantitative analysis of gene expression signatures was conducted for PG2 by hierarchical clustering of correlation coefficients. The results showed that PG2 product batches were consistent and of high quality. These batches were also functionally equivalent to each other with regard to how they modulated the immune and hematopoietic systems. Within the PG2 signature, there were five genes associated with doxorubicin: *IL-8, MDM4, BCL2, PRODH2*, and *BIRC5*. Moreover, the combination of PG2 and doxorubicin had a synergistic effect on induced cell death in HL-60 cells. Together with the bioinformatics-based approach, gene expression profiling provided a quantitative measurement for the quality and consistency of herbal medicines and revealed new roles (e.g., immune modulation) for PG2 in cancer treatment.

## 1. Introduction

Traditional Chinese medicine (TCM), also called botanical medicine or phytomedicine, is now widely recognized worldwide and is used to improve health and heal diseases. The therapeutic effects of TCM have been reported for various diseases, including liver disorders [1], hypertension [2], and cancer [3].* Astragalus mongholicus* has a long history as a traditional Chinese medicine, and it is used in the treatment of heart, liver, kidney, and immune system diseases [4]. Bioactive components of* Astragalus mongholicus* include polysaccharides, saponins, and astragalosides. Several studies have suggested that* Astragalus *polysaccharide (APS) might be used as an immunomodulator and could enhance immunological function [5, 6]. When used alone, APS had no anticancer activity in H22/ADM hepatoma cells* in vitro*. However, APS could enhance the sensitivity of hepatoma cell lines to chemotherapeutic drugs such as cyclophosphamide, doxorubicin, 5-fluorouracil, cisplatin, etoposide, and vincristine. APS also downregulates* MDR1* mRNA expression and inhibits P-glycoprotein (MRP1) efflux pump function, which decreases the expression of the MDR1 protein [7].

Cancer immunotherapy has recently been considered to be a theoretically ideal anticancer strategy as it exploits the ability of the adaptive immune system to stimulate anticancer mechanisms and attack malignant cancer cells. Immunotherapy drugs are being evaluated for the treatment of a number of cancers, including renal cell carcinoma, leukemia, lymphoma, multiple myeloma, and melanoma [8]. Cell-based immunotherapy is also a primary target for cancer treatment. Several immune cell types, including B cells, cytotoxic T lymphocytes (CTLs), dendritic cells (DC), natural killer cells (NK cells), and lymphokine-activated killer cell (LAK), are activated* in vivo* by cytokines, including interleukin-2 (IL-2), interleukin-7 (IL-7), and interleukin-11 (IL-11). As a result, these cytokines could improve the patients' immune system target cancer cells.

Many botanical medicinal herbs, which have immunomodulatory and antitumor activities, may function as immunotherapy adjuvants. Some active compounds isolated from medicinal herbs, such as* Astragalus mongholicus*, have been shown to be immunostimulative and to possess antitumor potency [9].

PG2, a promising clinical product, contains a mixture of polysaccharides that were extracted, isolated, and partially purified from the roots of* Astragalus mongholicus*. Currently, PG2 has been suggested to play an important role in hematopoiesis and cancer-related fatigue. In one clinical trial, ninety patients with advanced cancer were enrolled in phase II/III, double-blind, randomly assigned, placebo-controlled clinical trials to evaluate the efficacy of PG2 on cancer-related fatigue. The results showed that PG2 relieves cancer-related fatigue in advanced cancer patients [10]. In addition, the integration of PG2 and chemotherapy has also been shown to improve quality of life in non-small cell lung cancer (NSCLC) patients with platinum-based chemotherapy by physical capacity and several quality of life indices. This includes treatment-related symptoms, such as fatigue, nausea, vomiting, pain, and loss of appetite [11].

With regard to the quality and consistency of herbal medicine, Wen et al. [12] have described how to study the quality of Si-Wu-Tang, a traditional Chinese medicine, by microarray experiments. In this study, a hierarchical clustering analysis by correlation coefficients was used to examine estradiol, ferulic acid, and Si-Wu-Tang, and the final microarray data were highly reproducible. Using signatures from gene expression profiles, this study also identified potential molecular mechanisms and indications for Si-Wu-Tang via a Connectivity Map [13]. This pathway analysis identified the NRF2-mediated oxidative stress response as the most significant pathway responding to Si-Wu-Tang treatment.

Because PG2 is composed of polysaccharides and is not a pure compound, gene expression profiling was used to examine the quality and consistency of PG2 from different product batches. The results suggest that different product batches of PG2 are highly similar in their expression profiles. Using the PG2 signature to perform pathway and network analyses, the results indicated that PG2 is primarily involved in immune-related pathways. Moreover, combining PG2 with the anticancer agent doxorubicin synergistically enhances cell death in HL-60 cells. Together, these observations suggest that PG2 may serve as an immune modulator to improve cancer treatment.

## 2. Materials and Methods

### 2.1. Microarray Sample Preparation

PG2 (provided by PhytoHealth Corp., Taiwan) is a botanically derived drug that is extracted, isolated, and purified from* Astragalus mongholicus*. The molecular weights of PG2 range between 20,000 and 60,000 daltons. The dominant polysaccharides in this product are *α*-1,4-linked glucans with varying degrees of branching at the 6 positions of the backbone residues. Other polysaccharides and glycoproteins present in this product are arabinogalactans, rhamnogalacturonans, and arabinogalactan proteins. In this study, HL-60 cells were treated with three different product batches of PG2, which were manufactured by validated standard operation procedures during the preparation of the raw materials, intermediates, and final products (in compliance with the GMP requirements). These were dissolved in water, designated 6J1, 6J2, and 9J1 (see Supplementary Table S1 in Supplementary Material available online at http://dx.doi.org/10.1155/2015/917345), and then subjected to microarray analysis using Affymetrix HG-U133 Plus 2.0 arrays. HL-60 cells were cultured in Iscove's Modified Dulbecco's Medium (IMDM, GIBCO) with L-glutamine and 25 mM HEPES buffer without sodium bicarbonate. HL-60 cells (1 × 10^6^) were seeded on a 100 mm dish with medium containing 10% serum. After 3 days, the HL-60 cells were treated with different batches of PG2 or the corresponding vehicle (water). The total RNA preparation was carried out according to the manual in the RNeasy Mini Kit (Qiagen, Valencia, CA) [14], with an extra on-column DNase digestion for sample preparation. Samples were verified to be free of DNA contamination, and RNA integrity was checked by gel electrophoresis. RNA samples for the microarray experiment were further checked with Agilent Bioanalyzer to assess sample integrity. The microarray experiment was performed in the genomic center of National Yang-Ming University.

The data set used to identify the differentially expressed genes in HL-60 cells (with and without PG2 treatment) consisted of 11 PG2-treated and 3 vehicle-treated control microarray datasets from three different experiments. Additionally, to examine the quality and consistency of PG2, several additional small molecules and herbal extracts were included in this study, including 15d-PGJ2 (15-delta prostaglandin J2 or 15-Deoxy-Delta12,14-Prostaglandin J2), clopidogrel, etoposide, EH (*Vitis ficifolia* var.* taiwaniana*), and Ac9 (*Antrodia Camphorata*). The protocols and reagents used for hybridization, washing, and staining were based on Affymetrix's instructions. For the microarray analysis, the raw fluorescence intensity data within the raw CEL files were preprocessed using the Bioconductor [15] Affy package for normalization (based on the Robust Multichip Average (RMA) algorithm with R language from Bioconductor). The log2-transform of the intensity in PG2-treated samples and the vehicle-treated samples were used as the expression value for each probe.

### 2.2. Cell Culture and Drug Treatment

Trypan blue exclusion was used to determine the total number of viable cells present. This test is based on the principle that live cells possess intact cell membranes that exclude certain dyes, such as trypan blue, whereas dead cells do not. HL-60 cells were treated with PG2 in the presence of doxorubicin. Cell suspensions were mixed with an equal volume of 0.4% trypan blue immediately before counting using a standard hemocytometer. Viable cells displayed a clear cytoplasm, whereas nonviable cells displayed a blue cytoplasm. THP-1 cells were first differentiated by exposure to 100 nM PMA for 24 hours. Cells were then activated by treatment with 100 ng/mL LPS and various concentrations of PG2 for 16 hours, followed by detection of cytokine production with ELISA.

### 2.3. Quantitative RT-PCR

Total RNA was isolated using the RNeasy Mini kit (Qiagen, Valencia, CA). RT-PCR reactions were performed using the TaqMan RNA reverses transcription kit (Applied Biosystems, Carlsbad, CA, USA) and the TaqMan Universal PCR Master Mix (Applied Biosystems) by following the manufacturer's protocols. The RT-PCR analysis used the following primers: TaqMan* IL-8 *(Hs00174103_m1),* MDM4* (Hs00159092_m1),* BIRC5* (Hs00153353_m1),* PTPN11* (Hs00275784_m1),* NFKB2* (Hs00174517_m1), and* ACTB* (Hs99999903_m1).

### 2.4. Evaluation of Drug Interactions

The interaction between PG2 and doxorubicin was analyzed by Jin's method [16] to determine whether the combination was additive or synergistic. The analysis was performed using the following equation: combination index (*q*) = *D*_1+2_/(*D*_1_ + *D*_2_ − *D*_1_ × *D*_2_), where *D*_1+2_ represents the effect when cells were exposed to a combination of drugs 1 and 2, and *D*_1_ and *D*_2_ represent the effects when each drug was used alone. *D*_1_ and *D*_2_ indicate PG2 and doxorubicin, respectively. A value of *q* > 1.15 indicates synergism, a value <0.85 indicates antagonism, and a value between 0.85 and 1.15 indicates an additive interaction.

### 2.5. Statistical Analysis

To understand whether the three different product batches of PG2 exhibited similar biological functions, the quality and consistency between two microarray expression profiles were illustrated by a heat map and scatter-plot. A scatter-plot is commonly used to determine the quality of an experiment, with each point corresponding to the expression of a gene between two microarrays. Next, a two-sample *t*-test (*P* < 0.01) and Bioconductor limma package [17] were used to identify statistically significant genes from PG2 versus vehicle experiments. These statistical testing procedures were carried out in a more exploratory manner without adjusting for multiple comparisons so that all potential candidates could be validated later. A total of 1,111 and 991 significantly regulated genes (referred to as the PG2 signature) were obtained from microarrays from three product batches of PG2 (Table 1, Supplementary Table S2/S3). All experimental results were expressed as the mean ± standard error. Levels of significance were evaluated by one-way analysis of variance (ANOVA) where *P* < 0.05 was considered to be statistically significant.

### 2.6. Cliques Analysis in a Protein-Protein Interaction Network

Protein-protein interactions (PPIs) and graph theory were used to examine the potential critical components from the PG2 signature after the PPI network was built using various signatures as described previously [18]. The functional subset of genes in the network was evaluated via the *k*-clique analysis based on the PPIs. The PPI data were collected primarily from ConsensusPathDB (CPDB) [19]. There are 106,642 two-protein interactions (release v.23) that integrate from 18 public resources for interactions in* Homo sapiens*, including Reactome, BioGRID, MINT, Intact, and Bind, among other well-known protein-interaction databases. Clique is an undirected graph with all vertices connected to each other by an edge. A *k*-clique is defined as a fully connected network of *k* nodes; in other words, each pair of nodes in *k* is connected by an edge.

### 2.7. Pathway and Chemical-Protein Interaction Analysis

Pathway enrichment analysis via the ConsensusPathDB (CPDB) human pathway database [19] was performed to identify statistically significant functional categories. Pathways exhibiting significant *P* values (*P* < 0.05) and lower FDR (*q* < 0.05) with at least 4 overlapping genes were retained. STITCH (search tool for interaction of chemicals) [20] is a public database that contains PPI and chemical-protein interaction information for 300,000 small molecules and 2.6 million proteins. STITCH allows exploration of known and predicted connections between chemicals and proteins based on confidence scores derived from experiments, databases, and the literature.

### 2.8. *In Silico* Drug Screening via Connectivity Map

Connectivity Map (CMap) [13] is a virtual drug-screening database based on gene expression profiles. The system uses a rank-based pattern-matching algorithm based on the Kolmogorov-Smirnov statistics to evaluate the degree of similarity or dissimilarity between query tags and profile tags. A strong positive enrichment score (similarities) indicates that the corresponding agent of that profile induces the expression of the query. For a PG2 query, the most significant similarity can thus be used to formulate a preliminary hypothesis about hidden connections between drugs and affected genes.

## 3. Results

### 3.1. Scatter-Plot Matrix and Correlation Map with Hierarchical Clustering Analysis to Evaluate the Quality and Consistency of PG2

**Figure 1:**
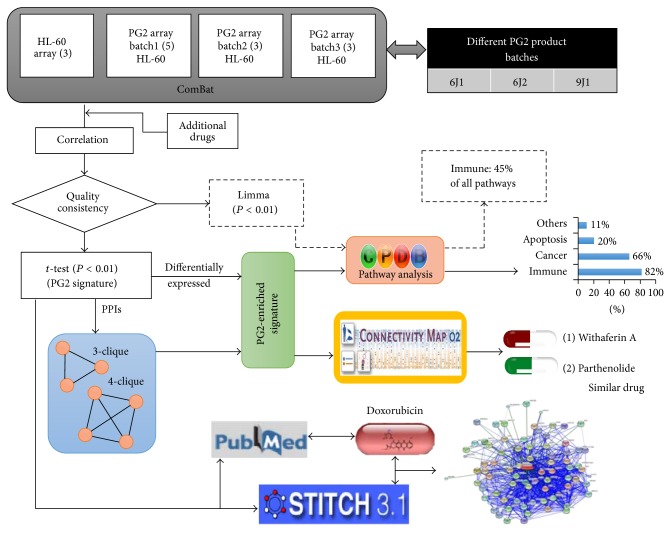
Schematic illustration of using PG2 gene signatures to infer PG2 quality and consistency, as well as the pathways induced by PG2. ComBat package was used to adjust the batch effect before analyzing expression profiles. To elucidate the consistency of three PG2 preparations, additional small molecule drugs and herbal extracts were included to test whether they are similar between each preparation according to correlation coefficient. Analysis of gene expression profiling, limma,* t*-test, topological clique, and fold-change cut-off analyses were used to identify the PG2-enriched signature. Chemical-protein interaction analysis via STITCH reveals a link between doxorubicin and PG2. Moreover, most of the immune-related pathways were retrieved from CPDB using the PG2-enriched signature. Finally, using the Connectivity Map tool, which used ranking by KS enrichment scores, two drugs were identified (withaferin A and parthenolide) that exhibit gene signatures similar to the PG2-enriched signature.

**Figure 2:**
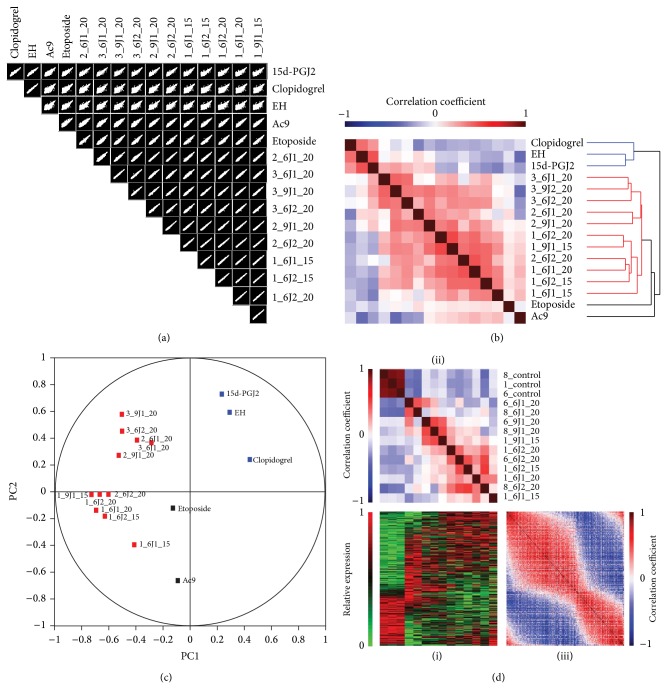
Scatter-plot matrix and correlation map with hierarchical clustering analysis show similarities between PG2 samples. (a) Scatter-plot matrix using all 54,675 probe sets for the gene expression profiles of HL-60 cells treated with PG2, 15-delta prostaglandin J2 (15d-PGJ2), clopidogrel,* Vitis ficifolia* var.* taiwaniana* (EH),* Antrodia Camphorata* (Ac9), and etoposide. All of the PG2 samples have very similar gene expression profiles because most of the data points fall very close to the 45° line. (b) The Pearson product-moment correlation matrix between the 16 samples, using relative expression for all 54,675 probe sets, is displayed by the GAP environment and sorted by the HCT_R2E algorithm. Both the sorted correlation matrix map and the dendrogram branching structure clearly indicate that all of the 11 PG2 samples form a cluster relative to the other samples. (c) The correlation plot of the first two components from the principal component analysis (PCA) of the same data reconfirms the findings in (b). (d) Three matrix maps (expression profile matrix (i), sample correlation matrix (ii), and gene correlation matrix (iii)) were calculated for the relative expression profile of 14 samples (3 vehicles and 11 PG2) with 646 upregulated genes and 465 downregulated genes, sorted and displayed by the GAP environment with the R2E (rank-two elliptical seriation) algorithm. The interaction pattern of the two sample groups (vehicle and PG2) on the two clear gene clusters (up- and downregulated genes) suggests that the PG2 signature is a well-defined gene set that can be used to analyze PG2.

To evaluate the quality and consistency of different PG2 product batches, the acute promyelocytic leukemia cell line HL-60 was chosen to serve as our model for the Affymetrix HG-U133 Plus 2.0 microarray analysis. In addition, several small molecules and herbal extracts were also included in the comparative analysis, including 15-delta prostaglandin J2 (15d-PGJ2), clopidogrel, etoposide,* Vitis ficifolia* var.* taiwaniana* (EH), and* Antrodia Camphorata* (Ac9).  Figure 1 shows a schematic flow chart summarizing the bioinformatics analysis. Scatter-plot matrix and correlation matrix maps with hierarchical clustering were used to study the consistency between samples from different preparations of PG2.  Figure 2(a) displays the 120 = C [2, 16] pairwise scatter plots of the gene expression profiles for the 11 PG2 samples, along with 5 small molecule drugs and herbal extracts. For two samples with identical gene expression profiles, all data points should fall on the 45° line, whereas samples with similar (different) gene expression profiles should have more (fewer) data points fall around the 45° line. As shown from the lower portion of Figure 2(a), all of the 11 PG2 samples have very similar gene expression profiles because most of the data points fall very close to the 45° line in related scatter plots. The wider scattering pattern of the data points in the scatter plots for PG2 than that of other drugs indicates a less similar relationship between these pairs of samples. Nevertheless, Pearson product-moment correlation coefficients for all 120 pairs of gene expression profiles ranged from 0.9774 (between EH and Ac9) to 0.9982 (between 1_9J1_15 and 2_6J2_20). This near perfect linear relationship always holds between two microarrays whenever the absolute expression level is used. For *i*th gene (probe set) (*i* = 1, …, 54675), the absolute expression *a*_*ij*_ (ranges from 2^0^ to 2^16^) between the 16 samples (*j* = 1, …, 16) is then converted into the within-gene relative expression *r*_*ij*_ (ranges from 0 to 1) by *r*_*ij*_ = (*a*_*ij*_ − *m*_*i*_)/(*M*_*i*_ − *m*_*i*_), where *m*_*i*_ = min⁡_1≤*j*≤16_(*a*_*ij*_) and *M*_*i*_ = max⁡_1≤*j*≤16_(*a*_*ij*_).  Figure 2(b) displays the correlation matrix map of the 16 samples using all of the 54,675 relative expression values (*a*_*ij*_, *i* = 1,…, 54675; *j* = 1,…, 16) and using the generalized association plot (GAP) environment [21, 22]. The HCT_R2E (average linkage hierarchical clustering tree with flipping of intermediate nodes guided by the rank-two elliptical seriation) algorithm in GAP was used to sort the 16 samples and to grow the dendrogram structure in Figure 2(b). Based on the dendrogram and the sorted correlation matrix map, the 16 samples were grouped into a cluster consisting of all 11 PG2 samples and a small but strong cluster of small molecules and herbal extracts (clopidogrel, EH, and 15d-PGJ2), with 2 individual samples of etoposide and Ac9 that were unclustered. A correlation-based principal component analysis (PCA) was then conducted on the same 16 samples with the 54,675 relative expression values and the correlation pattern of the first two principal components was plotted in Figure 2(c). All of the findings in Figure 2(b) were reconfirmed in Figure 2(c). Analyses shown in Figures 2(a)–2(c) indicated that different batches of PG2, although manufactured by the pharmaceutical industry at different times, retained similar quality for further scientific study.

### 3.2. Identification of PG2 Gene Signature

To identify the PG2 gene signature and further investigate its biological properties and functional mechanism, a two-sample* t*-test (*P* < 0.01) and the Bioconductor limma package were used to analyze the microarray data from the three PG2 product batches, and these analyses identified 1,111 and 991 statistically significant genes, respectively (Figure 1, Table 1, and Supplementary Tables S2 and S3). Following the procedures detailed in Figure 2(b), the relative expression matrix of the 1,111 significantly regulated genes in Figure 2(d)-(i) was first derived from their respective absolute expression as *r*_*ij*_ = (*a*_*ij*_ − *m*_*i*_)/(*M*_*i*_ − *m*_*i*_), (*i* = 1,…, 1111; *j* = 1,…, 14), where *m*_*i*_ = min⁡_1≤*j*≤14_(*a*_*ij*_) and *M*_*i*_ = max⁡_1≤*j*≤14_(*a*_*ij*_). The Pearson product-moment correlation coefficient matrix of these 14 samples and of the 1,111 statistically significant genes was calculated, as indicated in Figures 2(d)-(ii) and (iii), using the relative expression matrix from Figure 2(d)-(i). In Figures 2(d)-(ii) and (iii), the R2E (rank-two elliptical seriation) algorithm in GAP was used to sort both correlation maps for samples and for genes (probe sets), respectively. From these 3 permuted matrix maps, the interaction pattern of the two sample groups (vehicle samples and PG2 samples) on the two clear gene clusters (upregulated genes and downregulated genes) can be clearly identified. These findings suggest that the PG2 signature is a well-defined gene set that can be used to analyze PG2.

## 4. Construction of the PG2 Protein-Protein Interaction (PPI) Network to Reveal PG2-Enriched Signatures

To determine the functional connections between the 1,111 genes and to uncover the potential hubs from these differentially expressed genes, one possible approach is to conduct a protein-protein interaction (PPI) network analysis among queries [18]. As shown in Figure 1, several topological cliques in the PPI network were spotted and this information was used to address whether it was possible to identify pathways and similar drugs. Following the clique analysis, 3-clique and 4-clique sets consisting of 61 genes within the PG2 signature were identified as shown in Table 1 and Supplementary Table S2. Because the statistically significant genes with clique analysis from the gene expression profiles may not fully elucidate specific biological meaning, differentially expressed genes were also selected for functional mechanism analysis. Surprisingly, treatment with PG2 did not generate a significantly different gene expression pattern and a 1.3-fold criterion was used to select additional differentially expressed genes (27 upregulated and 5 downregulated) for further analysis. Thus, combining the set of differentially expressed genes with the gene set derived from the clique analysis revealed the PG2-enriched signature (65 up- and 22 downregulated genes), which was used for further analysis.

## 5. PG2 Signature Is Associated with Immune-Associated Pathway

Next, both the PG2-enriched signature and PG2-limma signature (Table 1) were used to perform pathway analysis via CPDB (Figure 1) [19].  Table 2 lists the top 30 enriched pathways for PG2. Using the PG2-enriched signature, a total of 76 statistically significant (*P* value < 0.05 and *q*-value < 0.05) immune-related pathways (approximately 82% of all results) were identified (Supplementary Table S4). On the other hand, there are 64 significant immune-related pathways (approximately 50% of all results) that were obtained from the PG2-limma signature (Supplementary Table S5). To illustrate the role of PG2 in the immune system, the PG2-enriched signature was processed by further analyses in this study. These enriched genes are involved in the control of the interleukin-family, B-cells, interferons, the TNF-family, and Jak-STAT signaling by regulation of the NF-*κ*B and JAK/STAT pathways. The combination of the NF-*κ*B and JAK/STAT pathways influences the production of different cytokines, which stimulates the proliferation and activation of different hematopoietic cell types.

**Figure 3:**
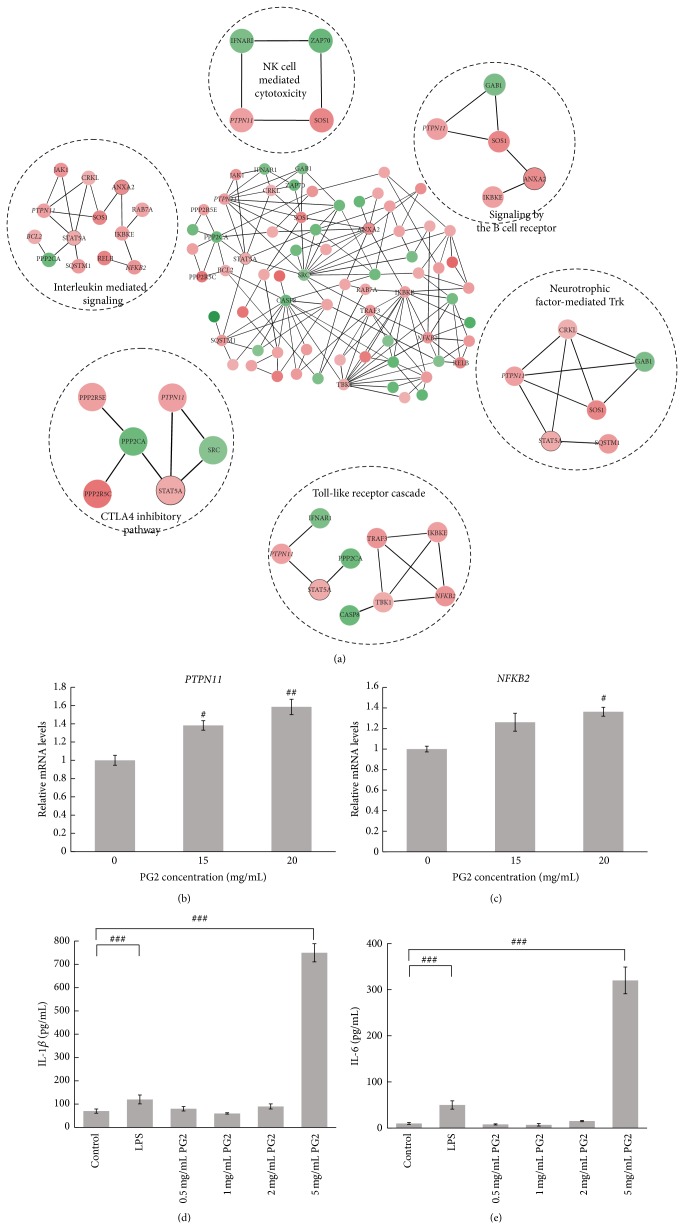
Network analysis of PG2-enriched signature reveals the connection to immune function. (a) The subnetwork was composed of 87 genes, including graph-based clique and overexpressed genes. These genes were subsequently analyzed using CPDB pathway analysis. As indicated in this figure, several subnetworks were identified, including several immune-related signaling pathways, such as interleukin-mediated signaling, toll-like signaling, Trk receptor signaling, and the CTLA4 inhibitory pathway. Links represent a protein-protein interaction in the network. The size of each node is based on the number of links to other nodes. Nodes with upregulated and downregulated genes are colored in red and green, respectively. The depth of color indicates the gene expression level fold change. Several genes from PG2-enriched signature, represented by a black borderline on a node (e.g., STAT5A in CTLA4 inhibitory pathway), are not in the original pathway analysis but are included in the pathways of the subnetworks based on a literature survey. The network was visualized using Cytoscape 3.0 (http://www.cytoscape.org/). HL-60 cells were treated with different concentrations of PG2 for 6 hr. (b)* PTPN11 *and (c)* NFKB2 *mRNA was measured by Q-RT PCR and normalized to *β*-actin (*n* = 3). PG2 treatment alone drastically increased the production of IL-1*β* (d) and IL-6 (e) in human THP-1 macrophages (*n* = 3) (^#^ *P* < 0.05; ^##^ *P* < 0.01; ^###^ *P* < 0.001).

Using the PG2-enriched signature to construct a PPI subnetwork, as shown in the center of Figure 3(a), six crucial immune response pathways were identified to be significant (*P* value < 0.05 and *q*-value < 0.05). These pathways included natural killer (NK) cell-mediated cytotoxicity, signaling by the B cell receptor, neurotrophic factor-mediated Trk receptor signaling, toll-like receptor cascades, the CTLA4 inhibitory pathway, and interleukin-mediated signaling (Figure 3(a)). Briefly, NK cells produce inflammatory cytokines and interact with other cells of the innate and adaptive immune system. NK cells also have antitumor functions as they efficiently attack target cancer cells that express low levels of MHC class I molecules [23]. Based on previous reports, *α*-galactosylceramide analogs, which are used as immunotherapy drugs, can induce IL-2 secretion to enhance the cytotoxic effects of NK cells [24, 25]. Importantly, activation of B lymphocytes (referred to as B cells) plays an important role in an efficient antitumor response by the immune system. Interestingly, some cytokines, such as TNF-*α*, IL-2, and TGF-*β*, can regulate synaptic plasticity and influence CNS functions. Microglia, which are immune cells found in the brain and spinal cord, can be detected in CNS injuries and have been found to produce and secrete a variety of neurotrophic factors, such as NGF, BDNF, neurotrophin-3 (NT-3), and neurotrophin-4/5 (NT-4/5). Microglia express members of the tyrosine kinase (Trk) receptor family, such as TrkA, TrkB, and TrkC [26]. Moreover, previous reports have shown that NT-3 increases microglial proliferation and phagocytosis through Trk receptor expression and stimulates microglial cells to attack cancer cells [27].

Recently, toll-like receptor (TLR) agonists have been identified to be potential candidates for cancer immunotherapy. TLRs induce the activation of innate immune responses that fight against invading pathogens by detecting conserved microbial components, such as lipopolysaccharides (LPS) and double-stranded RNA. TLRs have also been shown to encourage the activation of the immune system against cancer cells [28]. Cytotoxic T lymphocyte antigen-4 (CTLA4), which is expressed on activated T cells and a subset of regulatory T cells, is a potent negative regulator of T cell responses [29]. CTLA4 activation is mediated by its ligands B7-1 and B7-2 and results in decreased IL-2 transcription and T cell proliferation [30]. Blocking CTLA-4 activity can enhance antitumor T cell function and induce regression of breast carcinomas, lymphomas, and melanomas [31, 32].

The data showed that PG2 alone could drastically increase* PTPN11* and* NFKB2* expression in HL60 cells (Figures 3(b) and 3(c)) and the production of IL-1*β* and IL-6 in human THP-1 macrophages (Figures 3(d) and 3(e)), whereas the levels of IFN-*γ* and TNF-*α* were not altered by PG2 (Supplementary Figures S1A and S1B). These results confirm that PG2 alone can modulate certain types of immune system responses. Taken together, this bioinformatics analysis suggests that PG2 may augment the immune system and function as an immune modulator or immunotherapy drug.

## 6. Doxorubicin and PG2 Have a Synergistic Effect on Induced Cell Death of HL60 Cells

Immunotherapy regimens, such as IL-2 [33], IFN-*α* [34], and *α*-galactosylceramide (*α*-GalCer), have been approved for routine clinical use in oncology [35]. To develop an immune response, cancer immunotherapy is generally combined with chemotherapeutic drug treatment in cancer patients to enhance the sensitivity of tumor cells to chemotherapy [36, 37]. Furthermore, several upregulated genes (such as* HMOX1*,* GCLM*, and* NQO1*) affect oxidative stress (Supplementary Table S2). The antioxidant enzyme HO-1 (hemeoxygenase 1) is encoded by the* HMOX1*. HO-1 is a rate-limiting enzyme in the oxidative degradation of heme to biliverdin, free iron, and carbon monoxide (CO). It is induced by a variety of stimuli involved in cellular stress, including cytokines, reactive oxygen species (ROS), heat shock, hypoxia, and hyperoxia [38, 39]. Other transcripts elevated in PG2-treated cells include those for various ferritins, such as* FTH1* and* FTH1P5*, which are under the transcriptional control of NRF2, a transcription factor that may be activated under oxidative stress [40] and regulates the expression of many genes, including* FTH1*,* FTH1P5*,* GCLM*,* HMOX1*, and* NQO1*. Notably,* NQO1* is overexpressed after PG2 treatment. A previous report indicated that* NQO1* could increase the sensitivity of cells to chemotherapeutic agents such as cisplatin [41]. In this study, evidence was provided to show that the induction of genes encoding HO-1 and the ferritins is likely in response to cellular ROS generated via the NRF2 pathway upon treatment of HL-60 cells with PG2.

**Figure 4:**
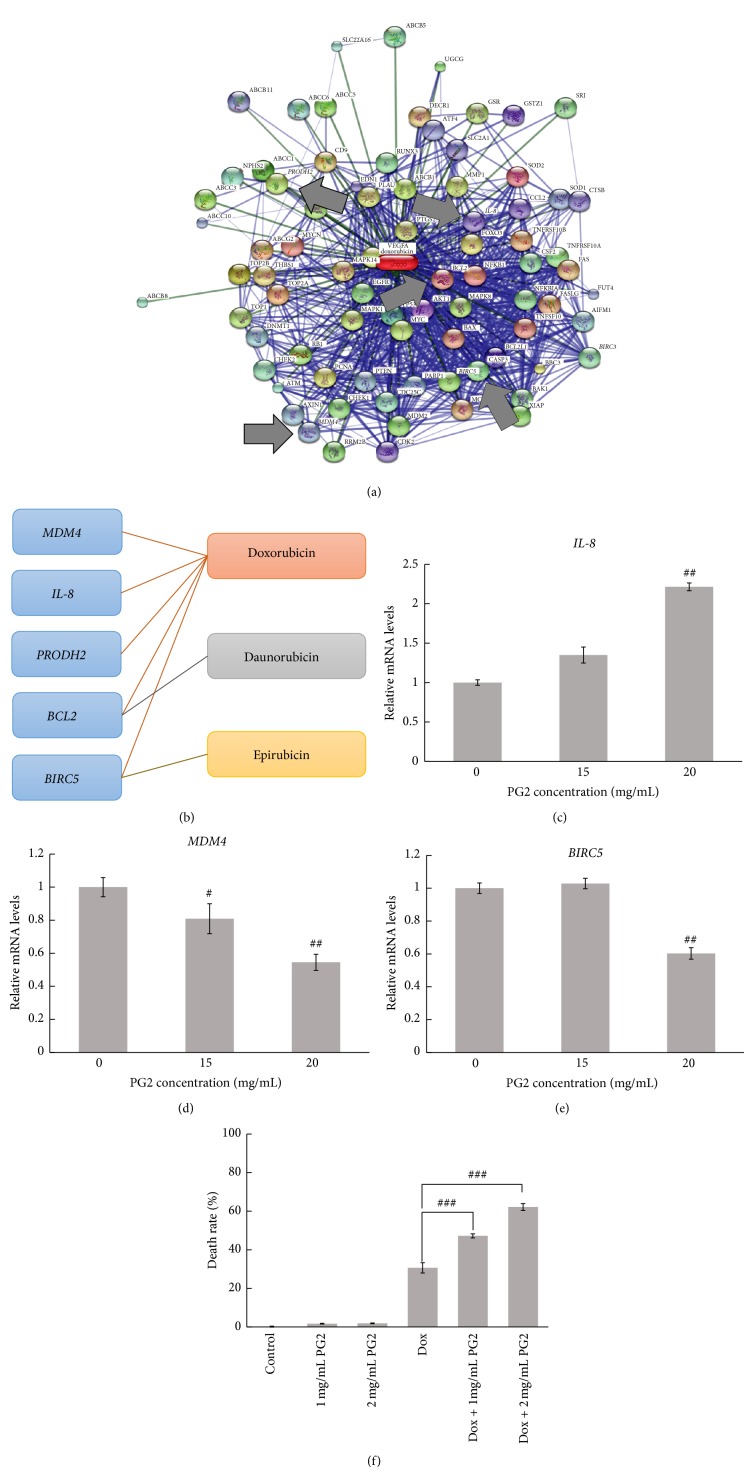
The effect of combining PG2 and doxorubicin on cell death in HL-60 cells. (a) The top 80 confidence interactions associated with doxorubicin were obtained from STITCH. Five of these (*MDM4*,* IL-8*,* PRODH2*,* BCL2*, and* BIRC5*) are derived from the PG2 signature and interact with doxorubicin (identified with arrows). Associated strength is represented by the thickness of the line. Protein-protein interactions are colored in blue, and chemical-protein interactions are colored in green. (b) Drug-target interactions between anthracyclines and PG2. Among these, daunorubicin and epirubicin are associated with only one interactor. HL-60 cells were treated with different concentrations of PG2 for 6 hr. (c)* IL8*, (d)* MDM4*, and (e)* BIRC5 *gene expressions were measured by Q-RT-PCR and normalized to *β*-actin (*n* = 3). (f) HL-60 cells were treated with various concentrations of PG2 in the presence or absence of doxorubicin (0.75 *µ*g/mL). Cell viability was determined by the trypan blue exclusion assay. Data are expressed as the mean ± standard deviation (SD) of three repeats. A representative result from three independent experiments is shown (*n* = 5) (^#^ *P* < 0.05; ^##^ *P* < 0.01; ^###^ *P* < 0.001 versus vehicle control group).

The anthracyclines are a class of antineoplastic drugs that damage DNA by intercalating into DNA strands in human cancer cells, particularly acute myeloid leukemia (AML) and acute lymphoblastic leukemia (ALL). Doxorubicin, daunorubicin, and epirubicin are anthracycline-based chemotherapeutic drugs and are most commonly used to treat these specific types of leukemia. In this study, STITCH was used to predict chemical-protein interactions between anthracyclines and the PG2 signature (Figure 1). Of these chemotherapeutic agents, doxorubicin had more interactions associated with PG2 than any other (Figures 4(a) and 4(b) and Supplementary Table S2). Additionally, multiple genes in the PG2 signature are associated with doxorubicin, including* IL-8*,* MDM4*,* BCL2*,* PRODH2,* and* BIRC5*. In addition to acute leukemia, doxorubicin is a well-known chemotherapeutic agent that is useful for treating various cancers, including non-Hodgkin's lymphoma, Kaposi's sarcoma, Ewing's sarcoma, Wilms' tumor, and breast cancer. Recent studies have shown that doxorubicin induces NF-*κ*B through interleukin-8 (IL-8) [42], whereas p53 inactivation is associated with the overexpression of MDM2/MDM4 in AML cell lines [43]. MDM4 overexpression is associated with a poor prognosis in chronic lymphocytic leukemia [44]. Moreover, previous study showed that* BIRC5* downregulation would help eradicate the chemotherapy resistant in ALL [45, 46]. Kuai et al. [47] reported that IL-8 plays an essential role in the adhesion, migration, invasion, and chemosensitivity of human gastric cancer cells. Interestingly,* BIRC5* and* MDM4* were downregulated and* IL8* was upregulated in HL-60 cells treated with PG2 (Figures 4(c)–4(e)), which might enhance the response to chemotherapy.

Accordingly, the effect of PG2 in combination with doxorubicin on leukemia cells was investigated. HL-60 cells were treated for 48 hr with increasing concentrations of PG2 (1 and 2 mg/mL) or doxorubicin (0.75 *µ*g/mL), alone or in combination, and inhibition of cell proliferation was assessed using the trypan blue exclusion assay. After HL-60 cells were treated with 1 and 2 mg/mL PG2 for 48 hr, the percentage of dead cells was 1.69% and 1.79%, respectively. Cell viability in cells treated with 0.75 *µ*g/mL doxorubicin alone for 48 hr was 30.63%. After combining doxorubicin treatment with 1 and 2 mg/mL PG2 for 48 hr, cell death increased dramatically from 30.63% to 47.2% and 52.18%, respectively (Figure 4(f)). A significant difference was observed between the combined doxorubicin and PG2-treated group and the doxorubicin only group (*P* < 0.001). The results of these drug interaction analyses indicate that a combination of PG2 and doxorubicin for 48 hr has a synergistic effect (*q* = 1.48 and 1.64) [16]. Therefore, the dose of doxorubicin could be reduced if combined with PG2 cotreatment, lowering the acute cardiotoxicity and increasing doxorubicin tolerance.

## 7. Search for Potential Small Molecules That Mimic the PG2 Mechanism 

A gene pattern-matching tool, Connectivity Map (CMap), was utilized to identify small molecules that have a strong association with PG2 by searching for highly similar gene expression patterns (Figure 1). For this analysis, 87 enriched genes (65 up- and 22 downregulated genes from the PG2-enriched signature) were used as the input query. The results of the Kolmogorov-Smirnov test in the CMap analysis are shown in Supplementary Table S6. Additionally, 1,000 samples of sizes 100, 200, 300, 400, and 500 probe sets were randomly sampled from the Affymetrix U133A gene signature (22,277 probe sets) to test the robustness of the CMap. Supplementary Table S6 and Supplementary Figure S2 show the drug ratio of 5,000 random results from this sampling.

The top 5 small molecules from the Kolmogorov-Smirnov enriched score were identified to have a similar gene expression pattern to that of PG2 via CMap. Of the top similar drugs, there are two small molecule drugs, withaferin A and parthenolide (Figure 1), whose gene signatures in CMap show a significantly (*P* value = 0) positive correlation (enrichment score equals 0.958 and 0.957, resp.) with the gene signature of PG2. Interestingly, both potential drugs have a near 10% random sampling ratio (the largest random sampling ratio from 1,309 CMap chemicals is approximately 30%). Therefore, based on the known mechanism of these similar drugs, PG2 is predicted to provide antitumor and anti-inflammatory effects in cancer patients and/or improve the effects of chemotherapy.

## 8. Discussion

Uncontrolled variation in potency between batches is problematic in herb extracts due to the use of raw botanical materials and the current manufacturing processes. The consistency of quality between batches needs to be controlled to ensure the efficacy and safety of botanical drug products for clinical applications. PG2 is a botanical drug composed of a mixture of* Astragalus* polysaccharides. In this study, established gene expression array profiling was used to explore the biological mechanisms of PG2 as well as the quality and consistency between different product batches of PG2 that were manufactured at different times. The microarray-based experiments were performed and analyzed at different times. Consequently, the resulting gene expression data might be influenced by nonbiological variables between different batches in the experiments. However, the common standard analysis procedure for adjusting the signal intensity of each microarray, referred to as normalization, is a nonspecific adjustment for removing nonbiological variables. For this reason, it is necessary to perform a batch effect correction on the microarray data analyses after normalization. Several approaches have been assessed for the removal of the batch effects. In this study, the adjustments were conducted using the Empirical Bayes algorithm “ComBat” [48] for multiple batches of experiments, which was demonstrated to be the most suitable for multiple batches and small sample sizes. Using this adjustment, it is possible to reduce the batch effects of experiments prior to analyzing gene expression profiles and evaluating the consistency between different product batches.

Next, the association of PG2 signature with many immune-related signaling pathways was demonstrated (Supplementary Table S4). The interleukin proteins are produced by leukocytes and play a key role in regulating the activation of immune responses for antitumor function and hematopoiesis [49]. The interleukin-family is involved in regulating the growth of blood cells and lymphoid cells, the development of bone-marrow cells, and the differentiation of T cells, B cells, and hematopoietic cells. Of these, activated T lymphocytes and B lymphocytes (referred to T cells and B cells) play an important role in the efficient antitumor response of the immune system. Tumor necrosis factor (TNF) is a cytokine produced by macrophages that can promote cancer cell death. The Jak-STAT signaling pathway mediates several biological functions, including the proliferation of immune cells [50]. A variety of cytokines, such as IFN-*γ*, IL-2, and growth factors, activate STAT factors by binding to cell surface receptors, which trigger the activity of receptor-associated Janus kinase (JAK) family members, including JAK1, JAK2, JAK3, and TYK2. Members of the JAK family play important roles in signaling downstream of cytokine receptor activation and are mutated in various hematological diseases, including cancer, through chromosomal translocations as well as point mutations [51]. This result is consistent with the findings of previous studies that demonstrated that PG2 can induce the differentiation and maturation of dendritic cells. It can further induce activation of immune function of T lymphocytes [52], restore the cytokine balance in the tumor microenvironment, and suppress the expression of* FOXP3* mRNA to inhibit the immune suppressive effects of T regulatory cells [53].

Our study demonstrated that PG2 might not only modulate the immune system but also increase the efficiency of cancer cell death by chemotherapeutic agents. Previous studies have shown that the regulation of apoptosis is subtly balanced between apoptosis-promoting factors (including TIA-1, p53, and caspases) and anti-apoptotic factors (BCL2, BIRC5 (Survivin), and MDM2). Many studies have shown that functional interactions between apoptosis-promoting factors play crucial roles in the management of cell growth and apoptosis. For example, p53 regulates the expression of MDM2 and BCL2. Moreover, BCL2 and BIRC5 (Survivin) are repressed by stabilization of p53 following DNA damage resulting from doxorubicin treatment in acute myeloblastic leukemia [54, 55]. As a traditional chemotherapeutic agent, doxorubicin acts mainly by breaking the DNA replication chain, which stops the process of replication and kills cells. Interestingly, several cell cycle-related pathways were downregulated after treatment with PG2, including DNA replication, M phase, Mitotic Prometaphase, and Mitotic M-M/G1 phases. Therefore, PG2 might be a potential agent to induce the sensitivity of chemotherapeutics in cancer, supporting our observation that PG2 exerts a synergistic effect when combined with doxorubicin.

Notably, a recent report indicated that the combination of certain drugs with doxorubicin enhanced the overall antitumor effects. For instance, metformin is a standard drug for treating type 2 diabetes and may inhibit cellular transformation and kill cancer stem cells [56]. It was demonstrated that the effect of simultaneous treatment with metformin and low doses of doxorubicin was better than treatment with doxorubicin alone. Another paper showed that combining quercetin with doxorubicin could reduce the DNA damage from doxorubicin and increase the effects in human breast cancer cells [57]. These aforementioned studies raise the possibility that combining a novel drug and a chemotherapeutic drug (doxorubicin) could increase their level effectiveness in cancer cells. In this study, PG2 has been shown to be able to enhance doxorubicin sensitivity in leukemia cancer cells and induce immediate activation of the cell cycle.

## Supplementary Material

Three different PG2 product batches were used to identify the gene signatures in this study.

## Figures and Tables

**Table 1:** Statistics for the PG2 gene signature from different product batches.

PG2 versus vehicle			
	PG2-6J1	PG2-6J2	PG2-9J1
Number of PG2 microarray	4	4	3
probe sets			
*t*-test			
Upregulated	284	533	332
Downregulated	122	369	227
Total number of probe sets	406	902	559
Excluded multiple or no gene symbol	307	666	418
		1,154	
Limma			
Total number of probe sets	388	1836	1272
Excluded multiple or no gene symbol	297	1272	936
		2,008	
Gene			
Limma			
Gene symbols	158	617	484
		991	
*t*-test			
Gene symbols	290	638	404
		1,111	
		Up: 646/Down: 465	
Differentially expressed genes	Up: 27/Down: 5		
*k*-clique gene symbols		Up: 65/Down: 22	
3-clique: 27 4-clique: 10	Up: 44/Down: 17		

A total of 11 PG2 microarrays were conducted in this study and divided into three product batches: 6J1, 6J2, and 9J1.

**Table 2:** Pathway analysis using the PG2-enriched signature (top 30 pathways).

Pathway	Source	#Genes in set	*p* value	*q*-value	Overlap	Immune	cancer
Immune system	Reactome	1011	2.16939*E* − 11	3.90490*E* − 09	28	□	
Pathways in cancer	KEGG	327	3.68916*E* − 09	3.32025*E* − 07	15		□
NF-kappa B signaling pathway	KEGG	92	1.01889*E* − 08	6.11336*E* − 07	9	□	□
Hepatitis C	KEGG	133	1.90274*E* − 08	8.56232*E* − 07	10		
Cytokine signaling in immune system	Reactome	186	4.29066*E* − 08	1.41014*E* − 06	11	□	
Signaling events mediated by stem cell factor receptor (c-Kit)	PID	53	5.95581*E* − 08	1.41014*E* − 06	7	□	□
RIG-I/MDA5 mediated induction of IFN-alpha/beta pathways	Reactome	53	5.95581*E* − 08	1.41014*E* − 06	7	□	
IL2 signaling events mediated by STAT5	PID	32	6.26727*E* − 08	1.41014*E* − 06	6	□	□
EPO signaling pathway	PID	34	9.20249*E* − 08	1.8405*E* − 06	6	□	
Epstein-Barr virus infection	KEGG	204	1.10634*E* − 07	1.99141*E* − 06	11		
Neurotrophic factor-mediated Trk receptor signaling	PID	65	2.52184*E* − 07	4.09444*E* − 06	7	□	
Measles	KEGG	134	2.72963*E* − 07	4.09444*E* − 06	9	□	
TRAF3-dependent IRF activation pathway	Reactome	13	1.33772*E* − 06	1.71993*E* − 05	4		□
Signaling events mediated by hepatocyte growth factor receptor (c-Met)	PID	84	1.48134*E* − 06	1.77760*E* − 05	7		□
Activation of IRF3/IRF7 mediated by TBK1/IKK epsilon	Reactome	15	2.52771*E* − 06	2.84368*E* − 05	4	□	□
MyD88-independent cascade	Reactome	97	3.91774*E* − 06	3.71155*E* − 05	7	□	
Toll-like receptor 3 (TLR3) cascade	Reactome	97	3.91774*E* − 06	3.71155*E* − 05	7	□	□
Beta-catenin phosphorylation cascade	Reactome	17	4.36224*E* − 06	3.92601*E* − 05	4	□	□
Platelet sensitization by LDL	Reactome	18	5.57985*E* − 06	4.78273*E* − 05	4	□	
Interferon signaling	Reactome	69	6.80349*E* − 06	5.56649*E* − 05	6	□	□
Signaling events mediated by TCPTP	PID	41	7.79283*E* − 06	6.03140*E* − 05	5	□	□
RIG-I-like receptor signaling pathway	KEGG	71	8.04187*E* − 06	6.03140*E* − 05	6	□	□
Chronic myeloid leukemia	KEGG	73	9.45705*E* − 06	6.65100*E* − 05	6	□	□
Activated TLR4 signaling	Reactome	111	9.60700*E* − 06	6.65100*E* − 05	7	□	□
Negative regulators of RIG-I/MDA5 signaling	Reactome	21	1.07467*E* − 05	7.16445*E* − 05	4		
Signaling by interleukins	Reactome	114	1.14527*E* − 05	7.36243*E* − 05	7	□	□
Inhibition of cellular proliferation by Gleevec	PID	22	1.30676*E* − 05	8.11090*E* − 05	4	□	□
Interleukin-3, interleukin-5, and GM-CSF signaling	Reactome	47	1.54549*E* − 05	8.74477*E* − 05	5	□	
CTLA4 inhibitory signaling	Reactome	23	1.57376*E* − 05	8.74477*E* − 05	4	□	
Neurotrophin signaling pathway	KEGG	120	1.60321*E* − 05	8.74477*E* − 05	7		
