# Supplementary material for: Gene Expression Profiling and Pathway Network Analysis Predicts a Novel Antitumor Function for a Botanical-Derived Drug, PG2
Source: Evid Based Complement Alternat Med. 2015 Apr 20;2015:917345. doi: 10.1155/2015/917345 (PMC4417974; doi:10.1155/2015/917345)
Supplement: Supplementary file 1 — Three different PG2 product batches were used to identify the gene signatures in this study. [file 917345.f1.zip › Supplementary Table S1S6 Figure S1S2.docx]

**Supplemental Tables and Figures**

**Supplementary Table S1. Detail information of 3 different PG2 product batches.**

| Experiment | Different PG2 product batches | Solvent | PG2 Concentration (μg/μl) | Cell line |
| --- | --- | --- | --- | --- |
| 1 | PG2-06J01 | Water | 15 | HL60 |
|  | PG2-06J01 | Water | 20 | HL60 |
|  | PG2-06J02 | Water | 15 | HL60 |
|  | PG2-06J02 | Water | 20 | HL60 |
|  | PG2-09J01 | Water | 15 | HL60 |
| 2 | PG2-06J01 | Water | 20 | HL60 |
|  | PG2-06J02 | Water | 20 | HL60 |
|  | PG2-09J01 | Water | 20 | HL60 |
| 3 | PG2-06J01 | Water | 20 | HL60 |
|  | PG2-06J02 | Water | 20 | HL60 |
|  | PG2-09J01 | Water | 20 | HL60 |

**Supplementary Table S6. Results of Connectivity Map analysis (top 5 list).**

| Drug name | n | enrichment | p value | *Random ratio | Mechanism |
| --- | --- | --- | --- | --- | --- |
| withaferin A | 4 | 0.958 | 0 | 11% | Anti-infective agent/Antineoplastic agent |
| parthenolide | 4 | 0.957 | 0 | 11% | Anti-inflammatory agent |
| 15-delta prostaglandin J2 | 15 | 0.59 | 0 | 12% | Anti-inflammatory Agent |
| lomustine | 4 | 0.917 | 0.00004 | 9% | Antineoplastic agent |
| calmidazolium | 2 | 0.991 | 0.00006 | 6% | Calmodulin antagonist |

*Random indicates random sampling tests in 5,000 times. Ratio represents the percentage of 5,000 tests and rank represents position of 1,309 drug list. High frequency means the probability of random selecting probesets for sampling via CMap.


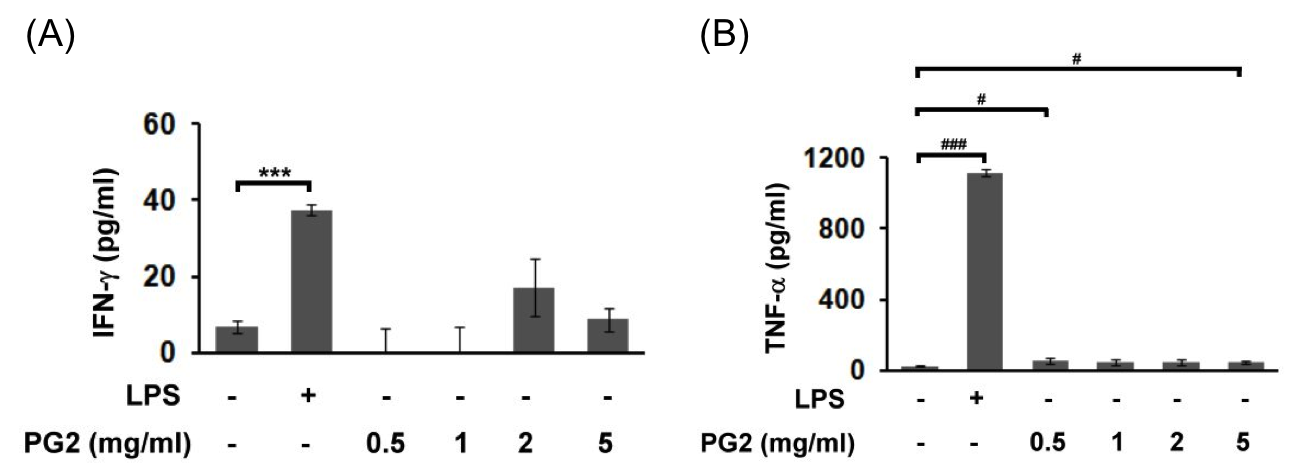


**Supplementary Figure S1: The levels of** IFN-γ and TNF-α were not altered by PG2.

**Supplementary Figure S2: The percentage of drug appearance by random sampling with different number of probesets in Connectivity Map**
